# Induced Pluripotent Stem Cells and Their Use in Cardiac and Neural Regenerative Medicine

**DOI:** 10.3390/ijms16024043

**Published:** 2015-02-13

**Authors:** Stepanka Skalova, Tereza Svadlakova, Wasay Mohiuddin Shaikh Qureshi, Kapil Dev, Jaroslav Mokry

**Affiliations:** Department of Histology and Embryology, Medical Faculty in Hradec Kralove, Charles University in Prague, Simkova 870, Hradec Kralove 50038, Czech Republic; E-Mails: skalova.step@gmail.com (S.S.); tereza.svadlakova@gmail.com (T.S.); wasay9710@yahoo.com (W.M.S.Q.); kapildchauhan@yahoo.com (K.D.)

**Keywords:** iPS cells, cell reprogramming, directed differentiation, Parkinson’s disease, Alzheimer’s disease, Huntington’s disease

## Abstract

Stem cells are unique pools of cells that are crucial for embryonic development and maintenance of adult tissue homeostasis. The landmark Nobel Prize winning research by Yamanaka and colleagues to induce pluripotency in somatic cells has reshaped the field of stem cell research. The complications related to the usage of pluripotent embryonic stem cells (ESCs) in human medicine, particularly ESC isolation and histoincompatibility were bypassed with induced pluripotent stem cell (iPSC) technology. The human iPSCs can be used for studying embryogenesis, disease modeling, drug testing and regenerative medicine. iPSCs can be diverted to different cell lineages using small molecules and growth factors. In this review we have focused on iPSC differentiation towards cardiac and neuronal lineages. Moreover, we deal with the use of iPSCs in regenerative medicine and modeling diseases like myocardial infarction, Timothy syndrome, dilated cardiomyopathy, Parkinson’s, Alzheimer’s and Huntington’s disease. Despite the promising potential of iPSCs, genome contamination and low efficacy of cell reprogramming remain significant challenges.

## 1. Introduction

Stem cells are unspecialized, self-renewing cells endowed with remarkable differentiation potential [1]. In 1908 Alexander Maximov used the term stem cell to describe the common precursor of the blood system [2] while their existence in bone marrow was first demonstrated in 1961 by James Till and Ernest McCulloch [3]. Another breakthrough in the field of stem cell research came in 1981 when Evans and Kaufman [4] isolated mouse embryonic stem cells (ESCs) from the inner cell mass of blastocysts (Table 1). It took more than a decade to isolate the first human ESC by Thomson *et al.* in 1998 [5]. Another remarkable year in stem cell research was 2006, when Yamanaka *et al.* reprogrammed adult mouse fibroblasts into induced pluripotent stem cells (iPSCs) using a set of defined transcription factors [6], which landed him the 2012 Nobel Prize in physiology and medicine. Later on in 2007, human somatic cells were also successfully reprogrammed into iPSCs [7].

**Table 1:** Stem cell timeline chart.

Year	Event
1908	The term stem cell was associated with haemopoiesis [2]
1961	Existence of stem cells in mouse bone marrow was demonstrated [3]
1981	Embryonic stem cell isolation from inner cell mass of mouse blastocyst [4]
1995	Embryonic stem cells isolation from rhesus monkey [8]
1998	Isolation of first human ES cells [5]
2006	Induced pluripotent stem cells from adult mouse fibroblast cells [6]
2007	Induced pluripotent stem cells from human fibroblasts [7]

## 2. Cellular Reprogramming and Induced Pluripotent Stem Cells

Discovery of the method for somatic cell reprogramming into iPSCs has transformed the field of stem cell biology and regenerative medicine [9]. In iPSC technology the pluripotent state is induced in mammalian somatic cells using a combination of ectopic expression of transcription factors [6]. The iPSCs are very similar in morphology, growth characteristics and genetic expression to ES cells [6]. History of cell reprogramming can be tracked back to the 1950s when Briggs and King established the method of somatic cell nuclear transfer (SCNT) and explored the developmental potential of nuclei isolated from late-stage embryos and tadpoles by transferring them into enucleated oocytes [10]. Work of Briggs, King and Gurdon led to the finding that differentiated amphibian cells can maintain the genetic information that is necessary to support the generation of cloned frogs [10,11]. The result was the development of a conserved, reversible epigenetic state, rather than irreversible genetic modification on the genome during cell differentiation [1]. SCNT enabled investigations into the developmental potential of cells [1]. In 1954, Stevens and Little established the immortal lines of pluripotent cells from testicular teratoma which remained undifferentiated *in vitro* [12]. Experiments with direct conversion of somatic cell to another type using transcription factor(s) (e.g., fibroblast to myoblast with MyoD [13]) paved the path to reprogramming cells to iPSCs.

ES cells and iPSCs have nearly identical phenotypes, including pluripotency marker expression, cell morphology, teratoma formation and differentiation into germ layers [14]. Similarity of the genome between pluripotent states of iPSCs can be compared with ESCs through knowledge of both the global chromatin structure and the gene expression programs [14]. However, some studies comparing the gene expression profiles of ESCs and iPSCs conclude that iPSCs are a unique cellular subtype, distinct from ESCs [14].

Induced pluripotent stem cells are characterized by expression of typical pluripotency markers like Oct4, Sox2, Klf4 and c-Myc [15]. Oct4 is a transcription factor that maintains the pluripotency and self-renewal of ESCs [16]. Reduced Oct4 expression leads to trophectoderm differentiation, while higher content potentiates differentiation into endoderm and mesoderm [17]. Oct4 function creates a heterodimer with Sox2 in ES cells, so that Sox2 binds to chromatin neighbouring to the Oct4 binding sites [18]. Sox2 is a part of the Sox gene family whose function is encoding transcription factors with a single HMG DNA-binding domain. Sox2 can maintain or preserve developmental potential of stem cells and is important for epiblast maintenance [19]. Klf4 is a member of the Kruppel-like factor family, also called a group of zinc finger, and the family contains transcription factors highly homologous with the Drosophila Kruppel protein. Klf4 plays an important role in regulating a diverse array of cellular processes including differentiation, development, proliferation, apoptosis and maintenance of normal tissue homeostasis [20]. c-Myc is a protein, which is the product of the c-Myc proto-oncogene and is a part of the processes of cell growth, cell proliferation, apoptosis and cellular metabolism [21]. The transcription factors c-Myc and Klf4 used in reprogramming are oncogenes [22].

The first iPSCs from adult mouse fibroblasts were reprogrammed by using the ectopic expression of four reprogramming factors Oct4, Sox2, c-Myc, and Klf4 (known as Yamanaka factors). These factors were introduced using retroviral vectors [6]. This reprogramming method effectively produces iPSCs but integrates with the genome resulting in insertional mutation. These risks were subsequently avoided with the introduction of modified methods, for example piggyBac transposon, Sendai virus, microRNAs, plasmid, episomal vector or minicircle vectors, but reprogramming efficiency still remains a substantial barrier [23].

The first murine and human fibroblasts were reprogrammed into iPSCs through over-expression of Oct4, Sox2, Klf4 and cMyc or Oct4, Sox2, Nanog and Lin28, but the low reprogramming efficiency remained the main obstacle [24]. Advances in iPSC technology solved long-standing problems of genome integration by exogenous introduction of reprogramming factors used as episomal plasmids [25]. During iPSC reprogramming, epigenome remodeling may facilitate such conversion of cell destiny by formation of cells more permissive to these epigenomic changes, such as Nanog and Lin28. This implies that compounds that alter cells epigenetics, for example, histone deacetylase, histone methyltransferase, histone demethylase or DNA methyltransferase, can improve the reprogramming efficiency or replace the use of certain transcription factors [26].

Several signalling pathways and chemical modulators, which serve to maintain pluripotency, may also be utilized during reprogramming to re-establish pluripotency. For example, Wnt pathway activation inhibits GSK3 (glycogen synthase kinase 3), leading to short-term self-renewal of mouse ESCs [27]. GSK-3 inhibitor (CHIR99021) may initiate reprogramming of mouse embryonic fibroblasts into iPSCs by over expressing only two factors, Oct4 and Klf4 [28]. When the factors were combined with GSK3 Parnate (also called tranylcypromine), inhibiting the lysine-specific demethylase 1, human primary keratinocytes were also reprogrammed by transduction of only two factors—Oct4 and Klf4 (first method of reprogramming human somatic cells without Sox2) [28] (see Table 2).

A second example is a TGF-β (transforming growth factor-beta) which induces growth arrest, tissue fibrosis and epithelial-mesenchymal transition by activating Smad and non-Smad signalling pathways [29]. TGF-β signalling inhibitor, RepSox, can replace Sox2 in cell reprogramming to pluripotency. It enables reprogramming over the induction of Nanog transcription in a permanent, partly reprogrammed cell type, which accumulates in the absence of Sox2 [30].

**Table 2:** Human and mouse iPSCs from different somatic cell types.

Species	Germ Layer	Cell Type	Reprogramming Factors	Reference
mouse	mesoderm	mouse embryonic fibroblasts	O, K, S, M	[6,31]
			O, K, S	[32]
		adipose-derived stem cells	O, K, S, M	[33]
		B lymphocytes	O, K, S, M	[34]
	endoderm	hepatocytes	O, K, S, M	[35]
			O, K, S	[35]
		pancreatic β cells	O, K, S, M	[36]
		gastric epithelial cells	O, K, S, M	[35]
	ectoderm	neural stem cells	O, K, S, M	[37]
			O, K, M	[38]
			O, K	[37]
			O, M	[37]
			O	[39]
human	endoderm	hepatocytes	O, K, S, M	[40]
	mesoderm	fibroblast	O, K, S, M	[6]
			O, L, S, N	[24]
			O, K, S	[41]
		mobilized peripheral blood	O, K, S, M	[42]
		peripheral blood and bone marrow mononuclear cells	O, K, S, M	[43]
		bone marrow stem cells	O, K, S, M	[42]
		circulating T lymphocytes	O, K, S, M	[43]
		umbilical endothelial cells	O, L, S, N	[44]
		cord blood stem cells	O, K, S, M	[42]
			O, S	[45]
		adipose-derived stem cells	O, K, S, M	[33]
		adipose stem cells	O, K, S	[46]
		mesenchymal stromal cells	O, K, S	[47]
		mesenchymal cells	O, K, S, M	[48]
	ectoderm	keratinocytes	O, K, S, M	[49]
			O, K, S	[49]
		neural stem cells	O	[39]
		melanocytes	O, K, M	[50]

O (Oct4), K (Klf4), S (Sox2), M (c-Myc), L (Lin28), N (Nanog).

Another method of human iPSCs preparation uses only Oct4. It applies a chemical cocktail (NaB, PS48, and A-83-01 and adds PD0325901 for week 5–8), which causes exogenous expression [51].

A highly effective method of reprogramming to pluripotency and directed differentiation of human cells is implied by using synthetic modified mRNA produced in *in vitro* transcription reactions templated by PCR amplicons. It was demonstrated that repeated administration of synthetically prepared mRNA, which contains modifications designed to bypass innate anti-viral responses, may lead to reprogramming of differentiated human cells into pluripotent ones with conversion effectiveness and kinetics significantly superior to established viral methods [52].

## 3. iPSC Differentiation into Three Germ Layers

The most important step for the iPSC application in diagnosis, therapeutic and regenerative medicine is to find appropriate methods that differentiate iPSCs into different cell types [53]. iPSCs, like other pluripotent stem cells, retain the ability to differentiate into all three germ layers [54]. *In vivo*, it is accomplished by teratoma formation, while *in vitro*, it is usually by embryoid body formation [40]. Upon providing specific microenvironmental clues, these cells continue to differentiate into different progeny to form terminally differentiated cells [53].

In the endodermal lineage, a great emphasis is given to hepatocyte differentiation [55]. Scientists have managed to prepare hepatocytes from iPSCs by combining Activin A, BMP4, and FGF2 in RPMI/B27 medium. Thus iPSC-derived hepatocytes can be used for liver disease modeling and regeneration [56]. Pancreatic cell differentiation mainly involves BMP/TGF-β inhibitors. Cho and colleagues used a combination of retinoic acid, FGF10, noggin (BMP inhibitor) and SB431542 (actin/TGF-β receptor antagonist) [55]. Other endodermal derivatives of iPSCs discussed in regenerative medicine are lungs and liver [57]. For example a combination of FGF2 and Sonic hedgehog (SHH) may lead to differentiation of the iPSCs into induced anterior foregut derivatives from definitive endoderm [58], while Wnt3a, FGF10, KGF, BMP4 and EGF generates lung cells [59].

The ectodermal differentiation of iPSC is mainly targeted towards neural lineage. Chambers and co-authors have differentiated iPSCs into neural cells with a help of FGF-2, ROCK-inhibitor, TGF-β inhibitor (SB431542) and Noggin [60]. This differentiation of iPSCs and its use in regenerative medicine is discussed in detail hereinafter in this review.

Cells of mesodermal origin include chondroprogenitor cells and blood cells that are important in regenerative medicine [61,62]. Guzzo *et al.* found that the direct plating of undifferentiated iPSCs at high cell density micromass cultures in the presence of BMP-2 leads to differentiation into chondroprogenitors [63]. Dias *et al.* prepared red blood cells using hiPS cells co-cultured with OP9 cells; these cells were then induced to mature in co-culture with MS5 cells that did not contain any cytokines. Factors that were used in differentiation were erythropoetin, thrombopoetin, interleukin 3, insulin, dexamethasone and iron-saturated transferrin [64]. The iPSC differentiation into mesodermal lineage is discussed in detail in the following section.

## 4. iPSC Differentiation into Cardiomyocytes

The most effective *in vitro* method for differentiation into cardiac muscle cells is by mimicking *in vivo* pathways that regulate the establishment of cardiac lineage during early development [65]. During early stages of cardiac differentiation, the cardiac mesoderm, can be induced by the temporal expression of KDR (Flk-1) [66]. Flk-1 encodes the vascular endothelial growth factor receptor VEGFR-2. Flk-1 is expressed in differentiating population of mesoderm cells and therefore, it relies solely on this receptor as a marker of early cardiac induction [67]. Further analysis of serum-induced murine ESC-derived population revealed the potential of cardiomyocytes to be characterized in Flk-1^+^ and PDGFR-α^+^ cell fraction [68].

Differentiation of pluripotent cells into cardiomyocytes is mostly triggered by formation of embryoid bodies using hanging drop method. Stage specific markers that encapsulate developmental processes in embryos, were identified including brachyury, in mesoderm cells (Mesp1, Flk1, and Pdgfra), in cardiac progenitors (Nkx2.5, Islet1) and in cardiomyocytes (myosin heavy chains). Developmental biology pointed to the possibility of differentiation of cardiomyocytes with growth factors such as Activin A/Nodal, Bmp4, Cerberus, Wnt3a, and Wnt11 [69]. Wnt11 plays an important role in the regulation of morphogenesis in several different tissues, including the heart by increasing the expression of cardiac marker genes, and acts as an important regulator of cell proliferation and differentiation during development of the myocardium [70]. Later, this method was combined with chemical biology, which used small molecules to control cell fate or modulate cell reprogramming [69].

Small molecules can serve as a complementary approach affecting specific signalling pathways, epigenetic regulators and also other cellular processes, and, thus, they provide influential tools for manipulating cell fate. A large number of these molecules can be used to maintain a self-renewal, to induce lineage differentiation and to relieve reprogramming by increasing the effectiveness of reprogramming or by substitution of genetic reprogramming factors [71]—see Table 3.

**Table 3:** Chemicals and small molecules used to differentiate iPSCs into cardiomyocytes.

Modifier	Name	Mechanism
Chemicals	Ascorbic acid	Enhances proliferation of CPCs via the MEK-ERK1/2 [72]
	Cardiogenol C	Activation of the Wnt signaling pathway and modified expression of several key chromatin remodeling proteins [73]
	Retinoic acid	Effects to growth factor stimulation pathway(s) [74]
	Szh-1	Unknown [69]
Small molecules	Pluripotin (SC1)	ERK1/Ras-GAP inhibition [75]
	RepSox	TGF-β receptor signaling inhibition [30]
	BIX01294	Histone methyltransferase inhibitor [76]
	Bay K 8644	Ca^2+^ channel agonist [77]
	RG108	DNA methyltransferase inhibitor [78]
	5-azacytidine	Inhibitors of DNA methyltransferases [79]
	Valproic acid	Histone deacetylase inhibitor
	SB431542	TGF-β superfamily type I activin receptor inhibition
	KY02112	Wnt inhibitor [80]
	DMSO	Decreases phosphorylation and increases levels of β-catenin [81]

Recently the cardiomyocytes were directly reprogrammed from mouse fibroblasts using different combinations of transcription factors, growth factors and miRNAs. The first method uses overexpression of cardiac transcription factors Gata4, Mef2 and Tbx5 [82]. The second method delivers miRNAs involved in cardiac development such as miRNAs miR-1, -133, -208 and -499 [82]. The last method involves over expression of reprogramming factors for iPS cells, which were Oct4, Sox2 and Klf4, and thereafter subjecting a small molecule inhibitor of the Janus kinase followed by culturing cells in cardiogenic media with the addition of bone morphogenic protein 4 (BMP4) [82].

## 5. iPSCs in Cardiac Disease Modeling and Regenerative Medicine

**Figure 1:**
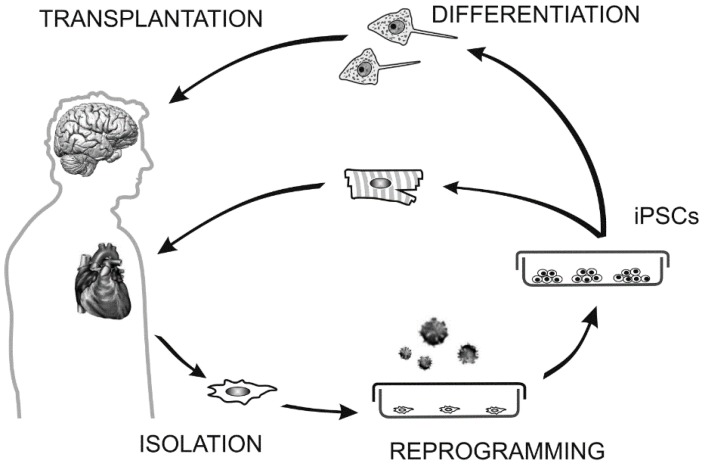
Generation of iPSCs and their use in cell transplantation.

During fetal development the cardiac muscle cells rapidly proliferate, but shortly after birth, the mammalian cardiomyocytes lead to termination of the cell cycle. Adult cardiomyocytes are capable of cell cycle re-entry, at a smaller scale and with the possibility of existence of species-specific differences [83]. However, it appears that a large number of cardiomyocytes predominantly grow postnatally by increasing cell size (hypertrophy), rather than number. This causes restrictions in the heart to restore function after any significant injury. Nevertheless, recent studies of cardiac progenitor cells capable of giving rise to cardiomyocyte-like cells led us to believe that the heart is a curable organ, and opens up new possibilities for regenerative medicine [83]. iPSC-derived cardiomyocytes could be transplanted to patients to repair their myocardium (Figure 1) or associated with acellularized scaffolds to create a bioartificial heart [84].

A wide variety of heart diseases are associated with a decrease in the number of functional cardiomyocytes, for example congenital malformations, such as hypoplastic and noncompaction syndromes, or acquired injury, such as exposure to cardiotoxic agents or injuries resulting from coronary artery disease, hypertension or surgical interventions. The restoration of cardiomyocytes in the heart is not sufficient enough to repair the damaged heart, so potential sources of donor cardiomyocytes, such as iPSCs and ESCs, for therapeutic intervention into damaged hearts, give us great hope. It was found that exogenous cardiomyocytes, transplanted into adult hearts, may integrate into the heart, both structurally and functionally [85].

Patient-specific somatic cells, fibroblasts, can be obtained from an easily accessible tissue, e.g., the skin. Isolation of viable cells is done with combination of enzymatic digestion and mechanical trituration. During *in vitro* cultivation the fibroblasts are reprogrammed with suitable transcription factors, e.g., Oct4, Sox2, Klf4 and c-Myc. A successful reprogramming yields colonies of iPSCs. If they bear a mutation causing a disease a defect can be genetically corrected *in vitro*. iPSCs can give rise to any cell type; directed differentiation *in vitro* can generate large amount of desired cells, e.g., neurons or cardiomyocytes. However, prior transplantation the remaining pluripotent cells have to be removed to prevent teratoma formation. Cardiac muscle or neuronal cells (or better the committed precursors of these cell lineages) are used in cell therapy to replace damaged or unhealthy cells from the patient organs. Such cell grafts are genetically identical to patient cells, thus reducing a risk of transplant rejection. Alternatively iPSCs can be used to seed the bioscaffolds to create artificial organs or to screen effects of new drugs.

In ischemic events, like myocardial infarction, or another disability, leading to a loss of cardiomyocytes, the self-regeneration of these cells is limited and the mechanisms that lead to the restoration of heart function usually include hypertrophy of surviving cardiomyocytes and proliferation of cardiac fibroblasts. This leads to irreversible heart failure, which requires heart transplantation but this is complicated by a lack of donors and the need for immunosuppressives to prevent rejection. New methods of treatment, using iPSCs and their differentiation into cardiomyocytes, give hope for circumventing complications related to heart transplantation [86].

The leading cause of death in the world population is myocardial infarction, an ischemic heart disease. Myocardial infarction leads to the loss of cardiac tissue, and subsequently to heart failure [87]. Due to the low potential to regenerate the heart, a scar forms after myocardial infarction [88]. Recent studies have shown that repair or regeneration of ischemic cardiac tissue may be accomplished by transplantation of functional cardiomyocytes to replace or repair injured myocardium [89].

Transplantation of pluripotent cells can be associated with risk of teratoma formation. After transplantation into the back of nude mice, Liu and his team monitored the proliferation and survival of undifferentiated iPSCs, iPSC derivatives and iPSC-derived cardiomyocytes. After killing mice they found teratomas of iPSCs and iPSC-derivates, but not iPSC-derived cardiomyocytes. The study demonstrated the ability of long-term existence of iPSCs during their differentiation *in vivo* [90]. To prevent tumorigenesis, undifferentiated pluripotent cells have to be removed from the graft prior to transplantation.

A suitable model for studying cardiovascular diseases can utilize iPSCs derived from the patient, in which the cardiac induction of these cell lines may simulate their respective disease pathophysiology *in vitro.* Cardiovascular disease, which includes long-QT syndrome, is mainly an autosomal dominant hereditary disease characterized by an abnormally prolonged ventricular repolarization phase [91]. Scientists thus have developed unique platforms for studying cellular and molecular mechanisms while assessing the efficacy of different drugs [92].

Timothy syndrome is a multisystem disease, causing tachycardia often leading to sudden death, is caused by mutations of the *CACNA1C* gene, the L-type calcium channel mutation [93]. Yazawa *et al.* examined the effect of this mutation on the contraction and electrical activity of human cardiomyocytes. They used human skin cells from a Timothy syndrome patient, which were reprogrammed into iPSCs and subsequently differentiated into cardiac muscle cells [94].

Other diseases, in which iPSCs have found their place, are forms of dilated cardiomyopathy. This disease is characterized by systolic dysfunction with normal LV wall thickness and ventricular chamber enlargement [95], caused by genetic mutation in troponin subunits, which is demonstrated by decreased Ca^2+^ sensitivity of force production [96]. iPSCs herein were useful as an important complementary model for generation of human cardiomyocytes to understand the physiological and cellular processes in dilated cardiomyopathy and also as a model for drug screening in human cells [97].

## 6. iPSC Differentiation into Neurons

The potential of iPSCs to differentiate *in vitro* into neural precursor cells and neurons has been reported [98]. It was demonstrated that neural differentiation of iPSCs is similar to that of human ESCs but with increased variability [99]. The iPSCs and ESCs form neural tube-like rosettes. However, iPSC-derived neuroepithelial cells were differentiated into regional progenitors and neurons in response to the same extracellular molecules, but the neural differentiation of iPSCs was variable and less efficient [99]. This low proliferative ability can be affected by the type of culture system or by adding extra neural inducers [99]. Due to the similarity in behavior of iPSCs and hESCs, similar factors can influence neural differentiation of these pluripotent cells. The use of retinoic acid (RA) with serum-free medium is a good example [98,100]. RA is a cell membrane-permeable morphogen that is already present during fetal development of the central nervous system. Its embryonic distribution correlates neural differentiation and positional specification [101]. In connection with that knowledge, it was demonstrated that RA concentration has an influence on neuronal differentiation of mouse ESCs. ESCs aggregated into the embryoid body (EB) when exposed to various concentrations of RA and show the precise expression profiles of neural and regional specific genes [102]. In EBs, a low concentration of RA (10^−8^ M) strongly promoted expression of nestin and Sox1, markers for neural progenitors [102]. EBs treated with higher RA doses (2 × 10^−6^ M) expressed low level of nestin but strong expression of β-tubulin III, typical for postmitotic neurons [102]. It follows that the lower concentration of RA preferentially induces undifferentiated neural progenitor cells from ESCs and a higher concentration induces differentiation of neural progenitors into postmitotic neurons and glial cells [102]. Ascorbic acid also plays an important role in neural differentiation. It is a vitamin with antioxidant properties that serves as a co-factor in several important enzyme reactions like dopamine- and glutamate-mediated neurotransmission [103]. It also increases the expression of genes involved in neurogenesis, neuron maturation, and neurotransmission [103,104]. It has been shown that ascorbic acid has a concentration-dependent effect on cell viability and development of dopaminergic neurons, either directly or through increased glial proliferation [105,106]. There exist protocols that involve typical soluble factors of neuronal development, like FGF2 or EGF and neural supplements like B27 or N2 [71,100,107,108]. Although, there are many promising results based on using these factors and molecules together with ESCs, situation with iPSCs could be different, so further studies are needed to confirm whether the findings will be similar.

## 7. Direct Differentiation into Neurons with Small Molecules and Their Properties

Efficient and homogeneous differentiation of human iPSCs to specific neural cells is an important step in neuronal disease treatment. There exist many protocols for differentiation of human ESCs and a few also have begun to appear for iPSCs (Table 4). However, the differentiation process usually concerns the development of particular cell lines frequently without individual, line-specific modifications. Small molecules seem to avoid this problem. As mentioned above, small molecules mimic the specific biochemical pathways and have many functions. However, specific methods using small molecules for neural differentiation of iPSCs, are less common [71,109]. One of the synthetic small molecules involved in neural differentiation and cell survival is ROCK inhibitor (Y27632). This molecule was used by Zhang *et al.* in a study dealing with Huntington’s disease cell model from iPSCs [100]. Menendez *et al.* used small-molecule compounds for Wnt signalling and a Smad pathway blockade for the efficient generation of self-renewing neural crest-like stem cells. CHIR, an inhibitor of GSK3-β, which participates in the Wnt signalling pathway, was used in this study to establish a role of this molecular pathway in neural crest cell formation [110].

Another highly effective method of fast neuronal differentiation into a homogeneous population of neuronal cells includes forced expression of a single transcription factor in ESCs or iPSCs. Zhang *et al.* used lentiviral delivery and tetracycline-inducible expression of exogenous proteins driven by a tetO promoter. Overexpression of neurogenin-2 or NeuroD1 (lineage-specific transcription factors) rapidly transformed ESCs and iPSCs into neuronal cells [111].

**Table 4:** Chemicals and small molecules used for neural differentiation.

Name	Mechanism
Retinoic acid	Morphogen/agonist of the Sonic Hedgehog pathway [102,112]
Epidermal growth factor (EGF)	Mitogen [113]
Fibroblast growth factor (FGF-2, FGF-8, FGF-4)	Regulation of neural stem cells proliferation and self-renewal [113]
Platelet-derived growth factor (PDGF)	Neural induction factor [113]
Sonic hedgehog (SHH)	Morphogen, induction factor [112]
Noggin	BMP antagonist [113]
SB431542	Inhibition of the TGFβ/Activin/Nodal pathway/inhibition of SMAD [114,115]
Dorsomorphin	Inhibition of BMP pathway/inhibition of SMAD [114]
LDN193189	Inhibition of BMP pathway [116]
Purmorphamine	Activation of the Hedgehog pathway [117]

## 8. iPSCs in Neuronal Disease Modeling

iPSCs have become a very important tool in neuroregenerative and degenerative disease research. Research on the human central nervous system and neurological diseases has been usually performed on post-mortem tissues or on animal models. In contrast iPSCs provide great potential to study human neurodegenerative and neurodevelopmental diseases in live neurons in a controlled environment. Thus, molecular mechanisms of the particular disease can be better studied due to the possibility of patient-specific somatic cells reprogrammed to iPSCs with a wide range of possibilities for early intervention and therapy [118]. The first study to show that human iPSCs can be used to model the specific pathology seen in a genetically inherited disease is that of Ebert *et al.* [119]. Ebert *et al.* found a disease phenotype is preserved in iPSCs derived from patients with spinal muscular atrophy and it selectively hinders motor neuron production and causes motor neuron degeneration during extended culture periods as a result of reduced expression of SMN (survival motor neuron) protein [119]. The use of iPSCs is, however, different *in vivo* than *in vitro* and carries some drawbacks, too. Diseases, in which increased neurotoxicity, due to sensitivity to oxidative damage and proteasome inhibition, may predominate over strictly synaptic deficits, are good examples. The iPSCs of these patients do not always exhibit the neuronal maturation and network defects *in vitro*, like in Parkinson’s disease and Alzheimer’s disease [120].

Currently, Parkinson’s disease is one of the most common neurodegenerative disorders, which is characterized with dopamine deficiency in striatum, death of dopaminergic neurons in the substantia nigra, and the creation of Lewy bodies, which are protein aggregates containing α-synuclein [121,122]. The first description of a biologically relevant cellular phenotype of iPSCs indicated that generation of iPSCs carry the p.G2019S mutation in *LRRK2* (the most common Parkinson’s disease-related mutation). G2019S-iPSCs were differentiated into dopaminergic neurons and expressed increased levels of α-synuclein. These cells also showed increased sensitivity to hydrogen peroxide, 6-hydroxydopamine and MG-132 (proteasome inhibitor) [123]. Another study showed that midbrain dopaminergic neurons, derived from iPSCs lines from an SNCA (α-synuclein gene) triplication patient, produced double the amount of α-synuclein protein compared to control. Triplication of SNCA causes a fully penetrant, aggressive form of Parkinson’s disease with dementia because of α-synuclein dysfunction [124]. Mutations, linked with recessively inherited Parkinson’s disease, include parkin. Dermal fibroblast-derived iPSCs from Parkinson’s disease patients with parkin mutations showed greatly increased transcription of monoamine oxidases A and B when differentiated into midbrain dopaminergic neurons. It also led to elevated oxidative stress induced by dopamine oxidation [125]. Application of iPSCs derived from the twins’ fibroblasts helped to identify differences existing between monozygotic twins discordant for Parkinson’s disease [126].

Alzheimer’s disease (AD) is another common neurodegenerative disease. It is characterized by a severe, progressive dementia. One of the pathological phenomena is the formation of amyloid plaques in the brain due to oligomerization, aggregation and accumulation of amyloid β peptide. The establishment and analysis of iPSCs from patients with AD was reported. Yagi *et al.* first established familial AD patient-derived iPSCs and confirmed that the production of highly toxic Aβ42 peptide is enhanced in all patient-specific iPSC lines. The finding strongly supports the amyloid cascade hypothesis, which holds that β-amyloid is the initiating factor in AD. In addition, iPSC-derived neurons carrying mutations responded sharply to γ-secretase inhibitors and modulators, indicating that neurons derived from patient-specific iPSCs hold tremendous potential in AD drug discovery research [127]. Subsequently, Israel *et al.* generated iPSC-derived neurons from familial AD, caused by duplication of the amyloid β precursor protein (APP) gene and two sporadic ADs and detected significantly higher Aβ40 levels. They also demonstrated other key pathological features, an increase of phosphorylated tau and its kinase, GSK activity in these AD-iPSC-derived neurons, showing a relationship between aberrant APP processing and tau [128]. In several studies it was observed that neurons derived from AD iPSCs exhibited increased vulnerability to glutamate-mediated cell death [129] and the accumulation of Aβ oligomers induced ER and oxidative stress leading to apoptosis [130,131]. Kondo *et al.* found that neural cells, derived from a patient carrying the pathogenic APP-E693D mutation and a sporadic Alzheimer’s disease patient, produced intracellular accumulation of Aβ oligomers [130]. The APP-E693D mutation causes atypical dementia, in which no amyloid deposition is detected by positron emission tomography with a Pittsburgh compound-B radioprobe and no tau deposits are formed. Therefore, it is controversial whether this dementia represents AD or not.

Huntington’s disease (HD) represents neuronal disease associated with inadequate neuronal maturation, synaptic deficiency, and failed connectivity in early-onsets [120]. It is a neurodegenerative autosomal dominant-inherited disorder caused by abnormal expansion of CAG repeats in the huntingtin gene. Accumulation of polyglutamine in the affected brain, which is a key pathological feature, ranks HD among polyglutamine diseases. Results of mutation in the huntingtin gene are progressive motor dysfunction, cognitive decline, psychological problems, immunosuppression and ultimately death, caused by the death of neurons in striatum and brain atrophy. Neural cells derived from iPSCs of transgenic HD monkeys show nuclear inclusions, oligomeric mutant HTT (huntingtin) aggregates, and increased cell apoptosis, which are typical features of HD [132].

The HD iPSC consortium deals with CAG-repeat-expansion-associated phenotypes, where 14 iPSC lines from HD patients and controls were generated and characterized. iPSC-derived neural cells showed pathological changes in electrophysiology, metabolism, cell adhesion and cell death with both medium and longer CAG repeat expansions. A previous study had found that the length of the pathological CAG repeat remained the same during reprogramming after long-term growth *in vitro* and after neuronal differentiation. In addition, lysosomal activity increased in HD-iPSCs, compared to control iPSCs, both during self-renewal and in iPSC-derived neurons. Cellular stressors and brain-derived neurotrophic factor (BNDF) withdrawal predominately threatened the longer repeat lines [133,134]. Chae *et al.* performed comparative proteomic analysis among normal human ESCs, iPSCs and HD-iPSCs. They identified 26 up- and down-regulated proteins involved in different biological processes, including protection from oxidative stress, (e.g., superoxide dismutase 1 and peroxiredoxin) mostly in HD-iPSCs. Furthermore, the protein BTF3 (basic transcription factor 3) was up-regulated in HD-iPSCs, and activated ATM kinase (ataxia telangiectasia mutated kinase involved in DNA-damage-related apoptosis pathway), which led to activation of the p53-mediated apoptosis pathway. On the other hand, the expression of cytoskeleton-associated proteins was down-regulated in HD-iPSCs. Overall, their results showed that HD-iPSCs are highly susceptible to oxidative stress, which leads to increased apoptosis and they also exhibit dysregulation of the cytoskeleton, influencing neuronal differentiation [135].

A deep insight into the pathogenesis of other polyglutamine diseases can be also obtained from *in vitro* models such as neural progeny of patient-specific iPSCs possessing disease-specific biochemical features with accumulation of polyglutamine protein. Using iPSC technology, Koch *et al.* gave evidence of l-glutamate excitation of neuronal cells of spinocerebellar ataxia type 3 (also known as Machado-Joseph disease) patients in initiation of ataxin-3 proteolysis that led to the formation of insoluble aggregates [136]. Nihei *et al.* reported that iPSCs derived from patients suffering from spinal and bulbar muscular atrophy are able to differentiate into motor neurons. Polyglutamine expansion occurs in patient-specific neurons following dihydrotestosterone treatment but not in iPSCs and fibroblasts, which confirms the neuron-dominant phenotype of this disease [137]. The authors found that 17-allylaminogeldanamycin sharply down-regulated the level of aggregates in neurons derived from patients’ iPSCs, demonstrating the potential of this model in pharmacological drug screening studies.

Human iPSC neurons reveal activity-dependent neurotransmitter secretion and can be advantageously utilized to study regulation of catecholamine biosynthesis related to CNS disorders with altered neurotransmission, like in schizophrenia [138]. iPSC lines obtained by reprogramming of fibroblasts from Friedreich’s ataxia patients retained genetic characteristics of the disease. iPSC derivatives differentiated into fully functional neuronal cells, had normal mitochondrial function and showed no altered susceptibility to cell death. Moreover, following neural grafting, the iPSC-derived neurons integrated well with the brain parenchyma [139].

## 9. iPSCs in Neuronal Diseases

Stem cell transplantation has always been a frequently discussed option for treatment of neurodegenerative diseases. Although there have been several clinical trials using human fetal neural cells for various diseases, results have been variable. Another problem is the limited availability of the fetal tissue, related ethical problems, and health risks due to incompatibility. Therefore, iPSCs seem to be a preferable, renewable and autologous source of cells for transplantation. Nevertheless, as already mentioned, the use of iPSCs is not without problems. Inadequate immune response in the formation of teratomas, generated from syngeneic iPSCs, is one of them [140]. However, recent studies found that syngeneic iPSC-derived cells indicate immunogenicity neither in culture nor after tissue engraftment and demonstrate minimal immune reaction against the teratoma tissue [141,142]. This raises important questions concerning the choice of vectors, methods and their subsequent standardization, testing of patients, *etc.* [120].

The therapeutic role of iPSCs may be a platform for drug discovery rather than for regeneration of diseased tissue. iPSCs offer many advantages over the traditional methods, which include preclinical studies mostly based on cell lines and animal models. Unlike these methods, which are limited by interspecies variations and their inability to fully recapitulate normal cellular function, iPSCs can provide a disease-specific, renewable source of human cells for more sensitive and accurate assessment of the tested compounds. iPSC-derived cell lineages can be also used for screening the effects of already known drugs [109,120]. As previously mentioned, there have been many studies on the potential of iPSC-derived specific-patient cell lines that offer not only modeling of molecular pathways, but also provide novel targets and a screening platform for the discovery of disease-modifying drugs. Studies focused on Parkinson’s disease have described the role of parkin in controlling dopamine utilization in human midbrain dopaminergic neurons and, among others, suppression of monoamine oxidase (MAO) by degrading estrogen-related receptors. Whereas the inhibition of MAO has modest, but significant effect in slowing down progression of Parkinson’s disease, it may be useful to mimic the protective function of parkin using inhibitors of estrogen-related receptors [125,141,143]. Another group of scientists focused on mitochondrial functions in Parkinson’s disease associated with mutation in the *PINK1* and *LRRK2* genes. They found that these iPSC-derived neurons are more sensitive to the chemical toxins valinomycin and concanamycin A than nervous cells from healthy subjects. Respecting that knowledge, iPSC-derived neural cells with these mutations were treated with the Coenzyme Q10, rapamycin or the *LRRK2* inhibitor GW5074 during exposure to low concentrations of either valinomycin or concanamycin A. The results suggest that cellular reprogramming technology can help to define groups of patients that react to different pharmacological treatments [144]. As for Alzheimer’s disease, Yahata *et al.* generated iPSC-derived neuronal cells, which expressed the forebrain marker Foxg 1 and neocortical markers CUX1, SATB2, CTIP2, and TBR1 and also amyloid precursor protein, β-secretase and γ-secretase components. Differentiated cells secreted Aβ into the conditioned medium and its production was inhibited by β-secretase and γ-secretase inhibitors and also sulindac sulfide, a non-steroidal anti-inflammatory drug. However, susceptibilities differed depending on stage of cell differentiation [145].

*In vivo* reprogramming would be a great alternative to cell-replacement therapy. It includes mobilizing resident cells already present in the target tissue to regenerate and repair the damage. One of the recent studies was designed to perform direct neural conversion *in vivo* by using transplanted human fibroblasts and human astrocytes. These cells were engineered to express inducible forms of neural reprogramming genes (complex-like 1 (*Ascl1*), brain-2 (*Brn2a*), and myelin transcription factor-like 1 (*Myt1l*)). These genes were activated after transplantation, and cells were converted into neurons afterwards. The authors of this study also found that endogenous mouse astrocytes can be directly converted into NeuN^+^ neurons *in situ* when they used a transgenic mouse model [146]. It is clear that development of *in vivo* reprogramming is still in the initial stage but it could open new dimensions in regenerative medicine.

Genetic correction of patient-specific iPSCs *in vitro*, and their subsequent transplantation is another option. Several molecular methods can be used for gene targeting to correct and introduce genetic mutations into the cell genome. Firstly, is homologous recombination technology, which was used in studies dealing with the genetic correction of HD. An *et al.* used genetic correction of iPSCs from HD patient fibroblasts, including the replacement of the expanded CAG repeat with a normal repeat. This correction persisted in iPSCs-derived DARPP-32-positive neurons *in vitro* and *in vivo* [147]. A second method of genetic correction uses helper-dependent adenoviral vectors [148]. A third method includes site-specific zinc finger nucleases (ZFNs), employed in repairing dominant A53T mutation in α-synuclein-associated PD [149]. Fourthly, RNA interference (RNAi) technology provides a new option for dominant negative genetic disorders, where a mutant allele of a gene causes disease in the presence of a second one. High mRNA target specificity and potency offers an effective opportunity to inhibit alleles of genes that show inherited or acquired polymorphisms, then also alternative or cryptic splicing with single point mutations. RNAi represents a new therapy for improvement of cardiac regeneration [150], genetic diseases, including amyotrophic lateral sclerosis, Alzheimer’s disease, Huntington’s disease, Parkinson’s disease, spinocerebellar ataxia, dominant muscular dystrophies, and also cancer [109,151].

In summary, though iPSC technology is not even a decade old, it has significantly revolutionized the world of stem cells, disease modeling, drug testing and regenerative medicine. The advent of improved methods to avoid insertional mutation has mainly overcome the shortcomings of using iPSCs in regenerative medicine. Although reprogramming efficiency remains low until now, it can be overcome by removing a reprogramming barrier or by releasing the somatic cells from a tightly locked epigenetic state before reprogramming.
